# Thermosensitive hydrogel containing dexamethasone micelles for preventing postsurgical adhesion in a repeated-injury model

**DOI:** 10.1038/srep13553

**Published:** 2015-09-01

**Authors:** Qinjie Wu, Ning Wang, Tao He, Jinfeng Shang, Ling Li, Linjiang Song, Xi Yang, Xia Li, Na Luo, Wenli Zhang, Changyang Gong

**Affiliations:** 1 State Key Laboratory of Biotherapy and Cancer Center, West China Hospital, Sichuan University, and Collaborative Innovation Center for Biotherapy, Chengdu, 610041, P. R. China; 2 School of Medicine, Nankai University, Tianjin, 300071, P. R. China

## Abstract

Tissue adhesion is a common complication after surgery. In this work, a dexamethasone loaded polymeric micelles in thermosensitive hydrogel composite (Dex hydrogel) was prepared, which combined the anti-adhesion barrier with controlled release of anti-adhesion drug. Dexamethasone (Dex) was encapsulated in polymeric micelles (Dex micelles), and then the Dex micelles were loaded into biodegradable and thermosensitive hydrogel. The obtained Dex hydrogel showed a temperature-dependent sol-gel-sol phase transition behavior. The Dex hydrogel could form a non-flowing gel *in situ* upon subcutaneous injection and gradually degrade in about 20 days. In addition, Dex hydrogel was assigned for anti-adhesion studies in a more rigorous recurrent adhesion animal model. Compared with normal saline (NS) and Dex micelles group, tissue adhesions in hydrogel and Dex hydrogel group were significantly alleviated. In Dex hydrogel group, the media adhesion score is 0, which was dramatically lower than that in blank hydrogel group (2.50, *P *< 0.001). In histopathological examination and scanning electron microscopy (SEM) analysis, an integral neo-mesothelial cell layer with microvilli on their surface was observed, which revealed that the injured parietal and visceral peritoneum were fully recovered without the concerns of adhesion formation. Our results suggested that Dex hydrogel may serve as a potential anti-adhesion candidate.

Postsurgical tissue adhesion is a common complication after surgery, which ranges from 60% to 90% after general surgical abdominal procedures^1^,^2^. Traumas (including mechanical injury, infections, ischemia, or presence of foreign bodies) to the peritoneum in the surgery are believed to be the reason of tissue adhesion^3^,^4^. The symptoms of postsurgical adhesion include chronic pain, female infertility, bowel obstruction, difficulties in re-operative procedure, and etc^5^,^6^. Postsurgical adhesion not only entails medical and economic burden to the patients, but also brings many troubles to surgeons^7^. Therefore, unremitting efforts should be made for preventing or alleviating postsurgical tissue adhesion^8^,^9^.

Previous studies proved that tissue adhesions were formed in the early period after surgery (generally in the first week)^10^,^11^. Therefore, researchers proposed to apply pharmacological or barrier approaches in the surgery to prevent the formation of adhesion^12^,^13^,^14^,^15^. Many pharmacological agents (including anti-inflammatory drugs, fibrinolytic drugs, and antibiotics) were used, but the therapeutic effects were limited, which may cause by the fast intraperitoneal clearance^16^. Anti-adhesion barriers were also widely studied, and the barriers generally fall into three categories, polymer solution, solid sheets, and cross-linking hydrogels^17^,^18^. However, due to their shortcomings, therapeutic effects of anti-adhesion barriers were not very exciting^19^. Thus, developing a safe and effective anti-adhesion agent is still a challenge.

In our previous works, poly(ethylene glycol)-poly(ε-caprolactone)-poly(ethylene glycol) (PEG-PCL-PEG) thermosensitive hydrogel was prepared, and the obtained hydrogel showed a special sol-gel-sol phase transition behavior. The PEG-PCL-PEG hydrogel was assigned for *in vitro* and *in vivo* toxicity evaluation, and the results suggested that the hydrogel could be used as a safe anti-adhesion barrier^20^. Then, the thermosensitive hydrogel was used to treat postsurgical tissue adhesion in several animal models, including sidewall defect-cecum abrasion model, cauterization-induced peritoneal adhesion model, and sidewall defect-uterine horn abrasion model^21^. After administration, the thermosensitive hydrogel could provide unrestricted coverage of the affected peritoneum. Then, the hydrogel converted into solid-like gel at body temperature and firmly adhered to the injured sites. The formed gel barrier separated the damaged surfaces, avoided formation of fibrinous adhesions between adjacent wounds, and eventually prevented tissue adhesion.

Then, we wonder if the thermosensitive hydrogel could still effective in a more rigorous recurrent adhesion animal model, in which the adhesion is more severe than side wall defect-cecum abrasion adhesion model and is more close to clinical conditions^22^. However, in our preliminary experiment, the hydrogel only showed limited effect. Previous contributions proved that anti-inflammatory drugs showed therapeutic effect on tissue adhesion, but the effects were compromised by short retention time. Therefore, in this work, we employed a dexamethasone loaded polymeric micelles in thermosensitive hydrogel composite (Dex hydrogel), which combines the anti-adhesion barrier with controlled drug delivery system, to treat the more rigorous rat repeated-injury adhesion model.

## Results and Discussion

### Preparation and characterization of dexamethasone loaded polymeric micelles (Dex micelles)

**Figure 1:**
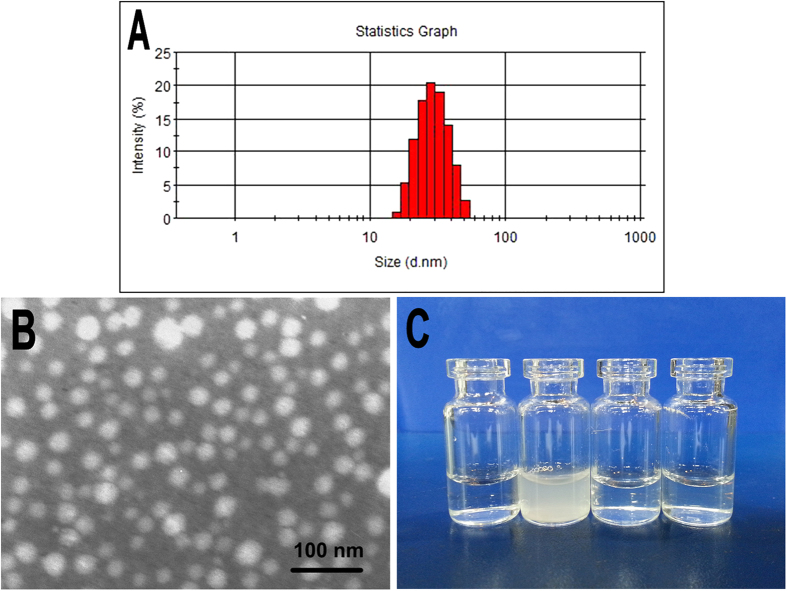
Characterization of Dex micelles. (**A**) Particle size of Dex micelles; (**B**) TEM image of Dex micelles; (**C**) Photograph of water, Dex in water, blank micelles, and Dex micelles (from left to right).

**Figure 2:**
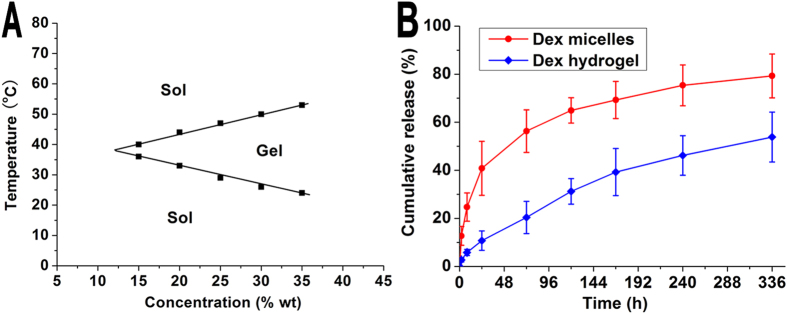
Phase transition and drug release behavior of Dex hydrogel. (**A**) Sol-gel-sol phase transition behavior of Dex hydrogel; (**B**) Release behavior of Dex from Dex micelles and Dex hydrogel.

Dex micelles were prepared using a one-step solid dispersion method. The encapsulation efficiency (EE) and drug loading (DL) of prepared Dex micelles were 1.90 ± 0.03% and 95.04 ± 1.60%, respectively. In addition, the particle size and polydisperse index (PDI) of the Dex micelles were 26.4 ± 1.8 nm and 0.129 ± 0.019, respectively (Fig. 1A). Figure 2B showed the transmission electron microscope (TEM) image of Dex micelles, and the spherical particles with small particle size about 25 nm were observed, which was consistent with the results of particle size analysis. Furthermore, the appearance of obtained Dex micelles was exhibited in Fig. 1C. Due to the hydrophobicility, Dexamethasone (Dex) formed a turbid suspension in water. In contrast, after encapsulated into polymeric micelles, Dex could form a clear and transparent solution.

### Preparation and characterization of Dex hydrogel

Owing to the hydrophilic PEG block and hydrophobic PCL block in PEG-PCL-PEG copolymer, aqueous solution of PEG-PCL-PEG copolymer displayed a special temperature-dependent sol-gel-sol transition. After Dex micelles were loaded into PEG-PCL-PEG hydrogel to form Dex hydrogel, sol-gel-sol phase transition temperature was determined. As shown in Figure 2A, when the concentration of PEG-PCL-PEG copolymer was above critical gelation concentration, the Dex hydrogel formed an injectable flowing sol at low temperature, and converted into a non-flowing gel at higher temperature. With increasing of copolymer concentration, the lower sol-gel transition temperature decreased, whereas, the higher gel-sol transition temperature increased. Concentration of 25% wt was chosen for the following experiments due to its appropriate sol-gel temperature.

*In vitro* release behaviors of Dex from Dex micelles and Dex hydrogel were investigated using a modified dialysis method. In comparison with Dex micelles, Dex hydrogel showed a much slower cumulative release behavior. As shown in Figure 2B, 10.74 ± 4.04% of Dex was released from hydrogel in the first 24 hours, which was significantly lower than that in Dex micelle group (40.84 ± 11.22%, *P *< 0.01). Furthermore, in two weeks period, 79.28 ± 9.12% of Dex was released from the micelles, whereas 53.84 ± 10.38% of Dex was released from the hydrogel (*P *< 0.01).

### *In vitro* cytotoxicity evaluation

**Figure 3:**
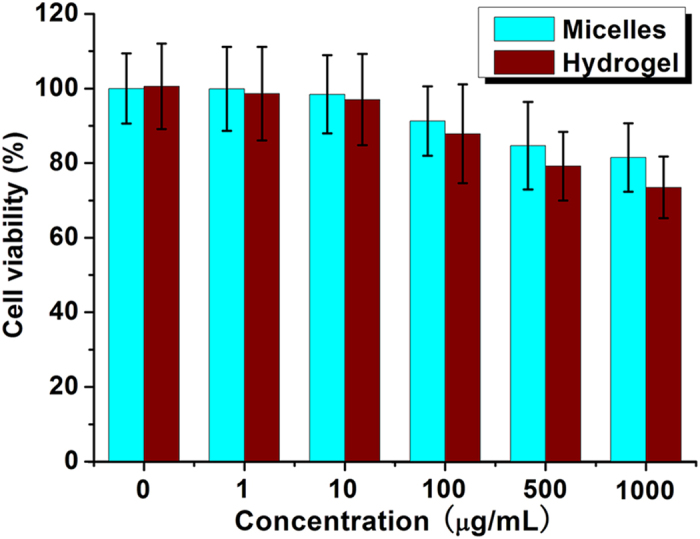
Cytotoxicity of blank micelles and hydrogel.

*In vitro* cytotoxicity of blank micelles and blank hydrogel was evaluated by cell viability on L929 cells using methyl thiazolyl tetrazolium (MTT) method. In Fig. 3, when the input concentration was 100 μg/mL, the cell viability of micelle or hydrogel group was 91.3 ± 9.3% or 87.9 ± 13.2% respectively, which suggested that the blank micelles and hydrogel showed low cytotoxicity. When the input concentration was even increased to 1000 μg/mL, the cell viability was still higher than 81.5% or 73.5% in micelle or hydrogel group, respectively. *In vitro* cytotoxicity tests indicated that the micelle and hydrogel could serve as safe drug carrier.

### *In vivo* degradation behavior

**Figure 4:**
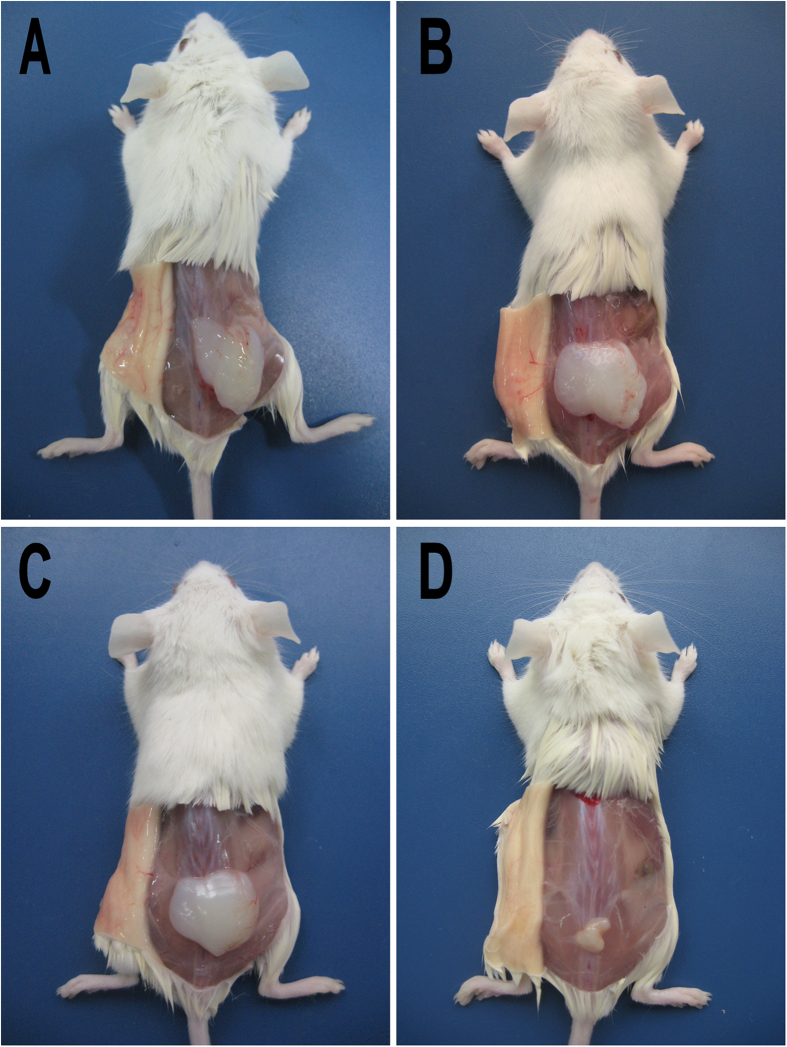
*In vivo* degradation behavior of Dex hydrogel. After administration for 1 (A), 5 (B), 10 (C), and 20 (D) days, mice were sacrificed, and the injection sites were opened to observe the state of Dex hydrogel.

As an anti-adhesion agent, *in vivo* degradation time is very important. Anti-adhesion agent should keep the injured tissue separated in the critical time of adhesion formation (about 7-14 days). *In vivo* degradation behavior of Dex hydrogel was assessed by subcutaneous injection in BALB/c mice. As shown in Fig. 4, Dex hydrogel formed a non-flowing gel upon subcutaneous injection. The Dex hydrogel became smaller over time, and disappeared after about 20 days. The *in vivo* degradation behavior of Dex hydrogel suggested it may serve as a potential anti-adhesion agent.

### Anti-adhesion efficacy of Dex hydrogel in rat repeated-injury adhesion model

In our previous works, the blank hydrogel was used to prevent peritoneal tissue adhesion in rat abdominal wall defect-cecum abrasion adhesion model, and showed a very exciting anti-adhesion effect. However, in our preliminary experiment, the hydrogel only showed limited effects on some more rigorous models such as repeated-injury adhesion model, which usually occurred in human surgery. Previous contributions proved that anti-inflammatory drugs showed therapeutic effect on tissue adhesion, but the effects were compromised by short retention time. Therefore, in this work, we employed Dex hydrogel, which combines the anti-adhesion barrier with controlled drug delivery system, to treat the more rigorous rat repeated-injury adhesion model.

**Figure 5:**
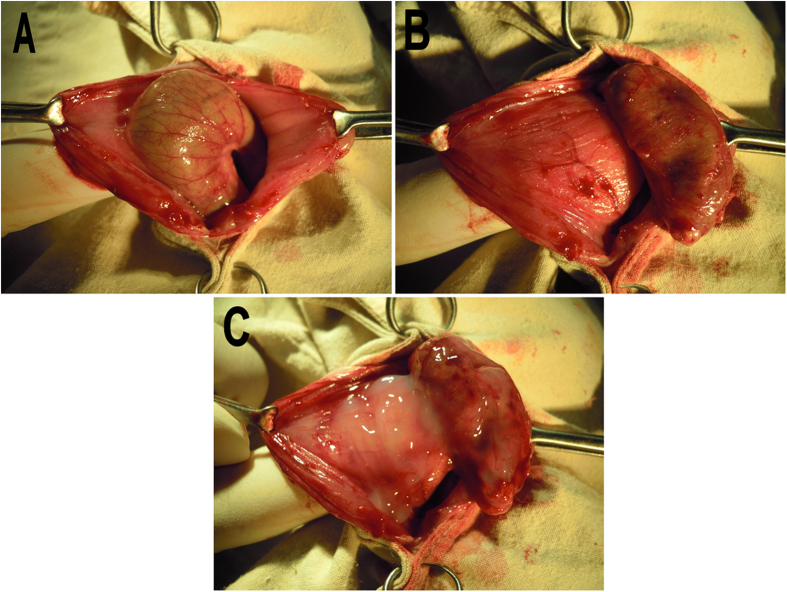
Establishment and treatment of rat repeated-injury adhesion model. (**A**) Abdominal wall defect-cecum abrasion peritoneal adhesion model was established; (**B**) One week later, a second laparotomy was performed, in which adhesions were cut by a blunt or sharp dissection as needed. The separated abdominal wall and cecal surface were re-abraded with a sterile brush until a bleeding surface was produced; (**C**) In Dex hydrogel group, 1 mL of Dex hydrogel was applied to coat the both damaged surfaces and the un-damaged surfaces around before complete closure.

**Figure 6:**
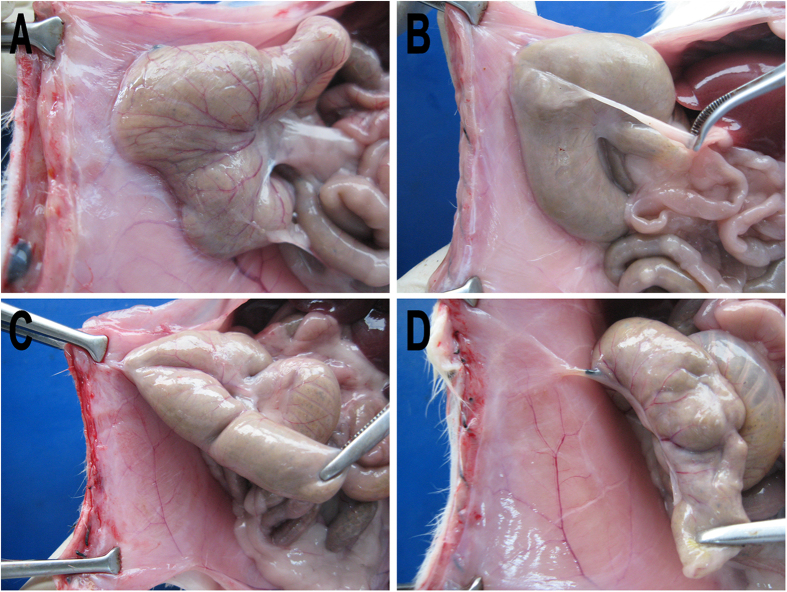
Evaluation of adhesion prevention in NS (A), Dex micelles (B), hydrogel (C), and Dex hydrogel (D) group.

Rat abdominal wall defect-cecum abrasion peritoneal adhesion model was established (Fig. 5A). One week later, a second laparotomy was performed, in which adhesions were cut by a blunt or sharp dissection as needed. The separated abdominal wall and cecal surface were re-abraded with a sterile brush until a bleeding surface was produced (Fig. 5B). Before complete closure, 1 mL of Dex hydrogel, Dex micelles, blank hydrogel or normal saline (NS) was applied to coat the both damaged surfaces and the un-damaged surfaces around (Fig. 5C). Then, two weeks later, the rats were sacrified to examine the anti-adhesion effects (Fig. 6 and Table 1). In NS group, 7 of 8 rats developed score 5 score adhesion, and the other one developed score 4 adhesion, which indicated that the repeated-injury adhesion model was established successfully (Fig. 6A). Dex micelles group only showed a very slight improvement in adhesion prevention, which may due to the fast absorption and metabolism of Dex micelles in abdominal cavity (Fig. 6B). In addition, compared with NS and Dex micelles group, tissue adhesions in hydrogel and Dex hydrogel group were significantly alleviated (Fig. 6C,D). In Dex hydrogel group, the media score is 0, which was dramatically lower than that in blank hydrogel group (2.50, *P *< 0.01, Mann-Whitney U-tests).

For examination of possible side effects, the mortality, body weight, and general conditions in each group were all monitored. In the whole experimental period, no accidental death occurred, and the body weight in each group showed no significant difference (data not shown). In addition, all the main organs in each group showed no apparent pathological changes. All these results indicated that Dex hydrogel is a safe anti-adhesion agent.

### Histopathological examination

**Figure 7:**
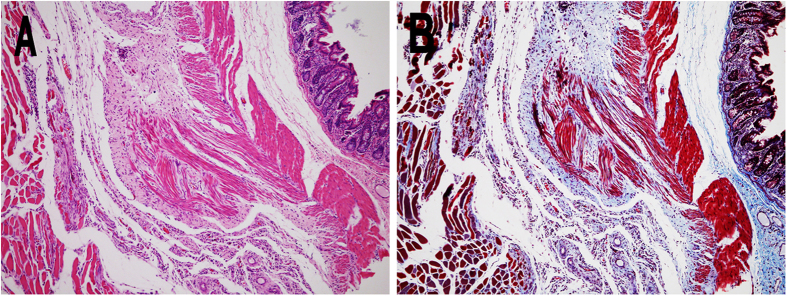
H&E (A) and Masson’s trichrome staining (B) of cross-section of an adhesion in NS group.

**Figure 8:**
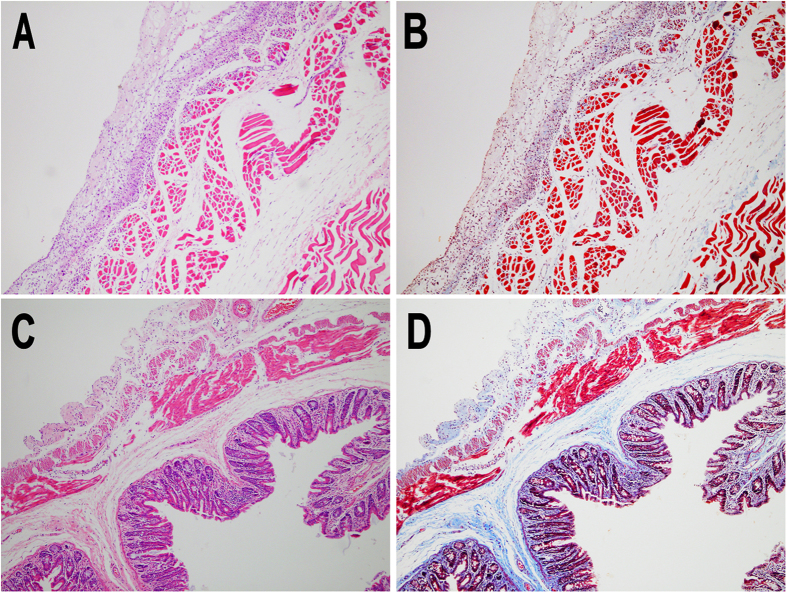
H&E and Masson’s trichrome staining of healed abdominal wall (A,B) and cecum (C,D) in Dex hydrogel group.

On histological examination, tissue samples from injured abdominal wall and cecum were stained with hematoxylin-eosin (H&E) and Masson trichrome staining (Fig. 7). As shown in Fig. 7, close apposition of the abdominal wall musculature and the smooth muscle layers of cecum were connected by various fibrous tissues in the tissues from adhesion sits in NS group, and the same condition was also observed in Dex micelles group (data not shown). In contrast, an integral neo-mesothelial cell layer with various subjacent fibrosis was observed in both abdominal wall and cecum in Dex hydrogel group (Fig. 8), which meant the injured abdominal wall and cecum were fully recovered without the concern of adhesion.

### The restoration of mesothelial cells layer of the peritoneum

**Figure 9:**
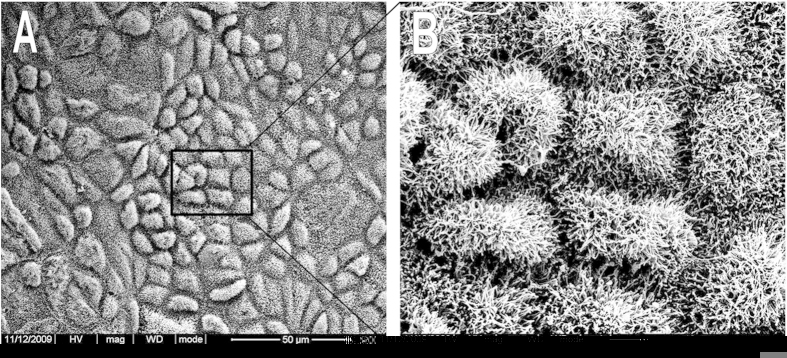
SEM image of surface of healed abdominal wall (A) and the image with higher magnification (B) in Dex hydrogel group. Microvilli on mesothelial cells were observed.

The recovery of parietal and visceral peritoneal injury and morphology of mesothelial cells were observed using scanning electron microscopy (SEM). Two weeks after the final administration, a layer of elongated, flattened, squamous-like cells located on the surface of recovered parietal peritoneum were observed in Dex hydrogel group (Fig. 9A), and the similar mesothelial cells were also observed in recovered visceral peritoneum (data not shown). Furthermore, in Fig. 9B, a carpet of microvilli on the surface of neo-mesothelial cells in parietal peritoneum occurred in a higher magnification of Fig. 9A. These findings revealed that the injured parietal and visceral peritoneum was fully recovered without the concerns of adhesion formation in the future.

## Materials and Methods

### Materials, cell line, and animals

ε-Caprolactone (ε-CL, Alfa Aesar, USA), monomethyl poly(ethylene glycol) (MPEG, Mn = 2000 and 550, Fluka, USA), hexamethylene diisocyanate (HMDI, Aldrich, USA), stannous octoate (Sn(Oct)_2_, Sigma, USA), dexamethasone (Sigma, USA), and 3-(4,5-dimethyl-2-thiazolyl)-2,5-diphenyl-2H-tetrazolium bromide (methyl thiazolyl tetrazolium, MTT, Sigma, USA) were used without further purification. All the materials used in this article were analytic reagent grade and used as received.

As described in our previous contributions, monomethyl poly(ethylene glycol)-poly(ε-caprolactone) (MPEG-PCL) copolymer was synthesized by ring-opening polymerization of ε-CL on MPEG using Sn(Oct)_2_ as catalyst, and poly(ethylene glycol)-poly(ε-caprolactone)-poly(ethylene glycol) copolymer was synthesized by ring-opening polymerization and coupling reaction using HMDI^23^,^24^. Molecular weight of synthesized MPEG-PCL or PEG-PCL-PEG copolymer was 3950 (2000–1800) or 3300 (550-2200-550), respectively (calculated by ^*1*^*H*-NMR).

L929 cells were purchased from the American Type Culture Collection (ATCC, Rockville, MD, USA), and grew in Roswell Park Memorial Institute 1640 medium (RPMI 1640, Gibco, USA) supplement with 10% fetal bovine serum (FBS, Caoyuan lvye, Huhht, China). The cells were maintained in a 37 °C incubator with a humidified 5% CO_2_ atmosphere.

Female Sprague Dawley (SD) rats (weighing 200 to 240 g) and female BALB/c mice (6–8 weeks) were purchased from HFK (Beijing HFK Bioscience Co. Ltd. China). All animal procedures were performed following the protocol approved by the Institutional Animal Care and Treatment Committee of Sichuan University (Chengdu, P.R. China).

### Preparation and characterization of Dex micelles

Dex micelles were prepared by a one-step solid dispersion method as reported^25^. In detail, MPEG-PCL copolymer and Dex were co-dissolved in ethanol, and evaporated into homogenous coevaporation. Then, the coevaporation was dissolved in NS to form Dex micelles.

Particle size distribution of Dex micelles was determined by a Malvern Nano-ZS 90 laser particle size analyzer. Morphology of Dex micelles was investigated using a TEM (H-6009IV, Hitachi, Japan). Concentration of Dex was determined by high performance liquid chromatography instrument (HPLC, Waters Alliance 2695), and EE and DL of Dex micelles were determined.

All results were the mean of three different samples, and all data were expressed as the mean ± standard deviation (S.D.).

### Preparation and characterization of thermosensitive hydrogel containing Dex micelles (Dex hydrogel)

The synthesized PEG-PCL-PEG copolymer was dissolved in NS (at the concentration of 25% wt) at designated temperature and cooled to 4 °C to form the biodegradable and thermosensitive hydrogel. Then, the prepared Dex micelles were added into the hydrogel to form the homogenous Dex hydrogel. Furthermore, the thermosensitive sol-gel-sol phase transition behavior of Dex hydrogel was investigated using a test tube-inverting method.

*In vitro* drug release behaviors of Dex micelles and Dex hydrogel were determined by a modified dialysis method^26^. Samples of Dex micelles and Dex hydrogel were placed in dialysis tube (molecular weight cutoff is 3.0 kDa), which were incubated in 10 mL of phosphate buffered saline (PBS, pH = 7.4) containing Tween80 (0.5% wt) at 37 °C with gentle shaking (100 rpm). At specific time points, all the release media were removed and replaced by pre-warmed fresh release media. Dex concentration in the released media was determined using HPLC. All results were the mean of three test runs, and all data were expressed as the mean ± S.D.

### Cytotoxicity evaluation

*In vitro* cytotoxicity of blank micelles or hydrogel was evaluated using MTT method on L929 cells. L929 cells were plated in 96-well plates and allowed to grow over night. Then, the cells were exposed to a series of blank micelles or blank hydrogel at different concentrations for 48 hours, respectively. The mean percentage of cell survival relative to that of untreated cells was estimated from data of six individual experiments, and all data were expressed as the mean ± S.D.

### *In vivo* degradation behavior

*In vivo* degradation behavior of Dex hydrogel was evaluated using BALB/c mice by subcutaneous injection. 500 μL of Dex hydrogel was administered by dorsal subcutaneous injection, and three mice were sacrificed at each time point (1d, 5d, 10d, and 20d). The injection sites were opened with a surgical scissors to observe the state of hydrogel.

### Treatment of rat repeated-injury adhesion model

Anti-adhesion efficiency of Dex hydrogel was evaluated in a rigorous recurrent adhesion animal model. Abdominal wall defect-cecum abrasion peritoneal adhesion model was established. In short, rats were anesthetized with a single intraperitoneal injection of 0.15 mL of pentobarbital sodium (50 mg/mL). An anterior midline incision was made through the abdominal wall and peritoneum and the cecum was identified and abraded until visibly damage by scrubbing with sterile dry surgical gauze. The damaged cecum was returned to the abdominal cavity, and a 1 × 1 cm apposing parietal peritoneal defect was created using sterile dry gauze. The abraded cecum was placed in apposition to the peritoneal wall defect, and the two damaged surfaces were approximated with 3/0 silk suture in order to induce adhesions as the cecum was too floppy in rats. Subsequently, the incision was closed in two layers with 5/0 surgical silk suture. One week later, a second laparotomy was performed, in which adhesions were cut by a blunt or sharp dissection as needed. The separated abdominal wall and cecal surface were re-abraded with a sterile brush until a bleeding surface was produced. Before complete closure, 1 mL of Dex hydrogel, Dex micelles, blank hydrogel or NS (8 mice per group) was applied to coat the both damaged surfaces and the un-damaged surfaces around. Animals were sacrificed with an overdose of intravenous sodium pentobarbital and examined for adhesion formation 14 days after last administration, by two observers in a blinded manner. Each animal was evaluated according to the following standard adhesion scoring system, which has been widely used in this field: score 0, no adhesion; score 1, one thin filmy adhesion; score 2, more than one thin adhesions; score 3, thick adhesion with focal point; score 4, thick adhesion with plantar attachment or more than one thick adhesion with focal point; score 5, very thick vascularized adhesion or more than one plantar adhesion.

### Histopathological examination

The damaged cecum, damaged abdominal wall, and adhesion tissues containing cecum and abdominal wall were collected and fixed in 4% paraformaldehyde in PBS for 72 h immediately. Then the tissues were embedded in paraffin, sectioned, stained with H&E and Masson trichrome staining under standardized conditions. All slides were analyzed using light microscopy (Olympus BX 45, Olympus, Hamburg Germany) by two pathologists in a blinded manner.

### SEM analysis of mesothelial cell recovery

SEM was employed to investigate the recovery of mesothelial cells on the surface of visceral and parietal peritoneum in Dex hydrogel group. Samples of damaged cecum and damaged abdominal wall were collected and fixed immediately with 2.5% glutaraldehyde in 0.1 M PBS. Then, the samples were dehydrated in a critical point apparatus, and were examined by SEM (JSM-5900LV, JEOL, Japan) after a gold sputter coating.

### Toxicity evaluation

Possible side effects of Dex hydrogel were evaluated. Rats were carefully observed after administration of Dex hydrogel, which included the mortality, body weight, and general conditions (including energy, activity, behavioral pattern, hair, feces, and other clinical signs). Furthermore, after treatment, main organs including liver, spleen, kidneys, lungs, and heart were harvested and fixed with 4% paraformaldehyde in PBS. Then, the obtained tissues were sectioned, stained with H&E and observed by two pathologists in a blinded manner.

### Statistical analysis

The statistical analysis was carried out using SPSS 15.0 software (Chicago, IL, USA). Adhesion scores did not always follow a normal distribution, therefore statistical inferences were made using Mann-Whitney U-tests, or Fisher’s exact test. A *P* value < 0.05 on a 2-tailed test was considered statistically significant.

## Conclusions

In this work, Dex hydrogel was prepared, which showed a temperature-dependent sol-gel-sol phase transition behavior and a controlled release behavior of Dex. *In vitro* cytotoxicity evaluation indicated that the micelles and hydrogel are biocompatible with low toxicity. After subcutaneous injection, the Dex hydrogel formed non-flowing gel *in situ* and gradually degraded in about 20 days. In a more rigorous recurrent adhesion animal model, Dex hydrogel significantly prevented the formation of adhesion, which combined the anti-adhesion barrier with controlled release of anti-adhesion drug. Dex hydrogel may serve as a potential anti-adhesion candidate.

## Additional Information

**How to cite this article**: Wu, Q. *et al.* Thermosensitive hydrogel containing dexamethasone micelles for preventing postsurgical adhesion in a repeated-injury model. *Sci. Rep.* **5**, 13553; doi: 10.1038/srep13553 (2015).

## Figures and Tables

**Table 1:** Adhesion prevention of Dex hydrogel in a rat repeated-injury adhesion model.

	Control(n = 8)	Dex micelles(n = 8)	Hydrogel(n = 8)	Dex hydrogel(n = 8)
Score 5	7	5	2	0
Score 4	1	1	1	1
Score 3	0	1	1	1
Score 2	0	1	2	0
Score 1	0	0	0	0
Score 0	0	0	2	6
Median score	5.00	5.00	2.50	0**

In comparison with NS, Dex micelles, and hydrogel group, treatment with Dex hydrogel significantly prevented adhesion formation (**P < 0.01).
